# Oral administration of recombinant *Bacillus subtilis* spores induces protective immunity against *Echinococcus granulosus* infection in dogs

**DOI:** 10.1186/s13071-025-07172-5

**Published:** 2025-12-01

**Authors:** Guoqing Shao, Xiaowei Zhu, Ruiqi Hua, Zhiwei Lu, Luo Wang, Zhuoyue Sun, Guangyou Yang

**Affiliations:** Department of Parasitology, College of Veterinary Medicine, Sichuan Agricultural University, Chengdu, 611130 Sichuan Province People’s Republic of China

**Keywords:** *Echinococcus granulosus*, Oral vaccines, *Bacillus subtilis*, Dogs, Immunity, Parasitic diseases

## Abstract

**Background:**

*Echinococcus granulosus* sensu lato, a cestode that inhabits the small intestines of canids, causes cystic echinococcosis (CE), a globally distributed zoonosis, through its larval stage. Vaccination is a cost-effective strategy to control *E*. *granulosus* infection in dogs. However, although dogs are the definitive hosts and main sources of CE transmission, no effective oral vaccines are currently available for them.

**Methods:**

Three *E*. *granulosus* proteins, enolase (EgENO), severin (EgSev), and cyclophilin (EgCyc), were selected as novel oral vaccine candidates. These proteins were fused to the CotB spore-coat protein and expressed on the surface of *Bacillus subtilis* spores. A cocktail vaccine comprising the three recombinant spores was orally administered to beagles. Two weeks after the booster immunization, each dog was challenged with 70,000 protoscoleces. At 21 days post-infection, necropsies were performed to assess the intestinal parasite burden and calculate the worm reduction rates. Fecal and serum samples were collected weekly to measure secretory IgA, IgG, and cytokine responses. Histopathological analysis of intestinal tissues was also performed.

**Results:**

The cocktail vaccine reduced intestinal *E*. *granulosus* colonization by 62.26% (*P* < 0.05) compared with *B*. *subtilis* 168 spore-only controls. Vaccinated dogs developed both mucosal and humoral immune responses against *E*. *granulosus* antigens. By day 14 post-boost immunization, serum cytokine profiling revealed that levels of IFN-γ, IL-4, and IL-10 in the vaccinated group were significantly higher than those in the control group (*P* < 0.05). Histopathological analysis confirmed that the vaccine caused no adverse effects and alleviated the intestinal damage induced by the parasite.

**Conclusions:**

This study demonstrates that *B*. *subtilis* spores serve as a safe and effective bacterial carrier to deliver *E*. *granulosus* antigens, supporting their potential in protecting dogs against *E*. *granulosus* infection. The heterogeneity in immune responses among vaccinated dogs should be addressed in future studies to secure consistent herd-level protection.

**Graphical abstract:**

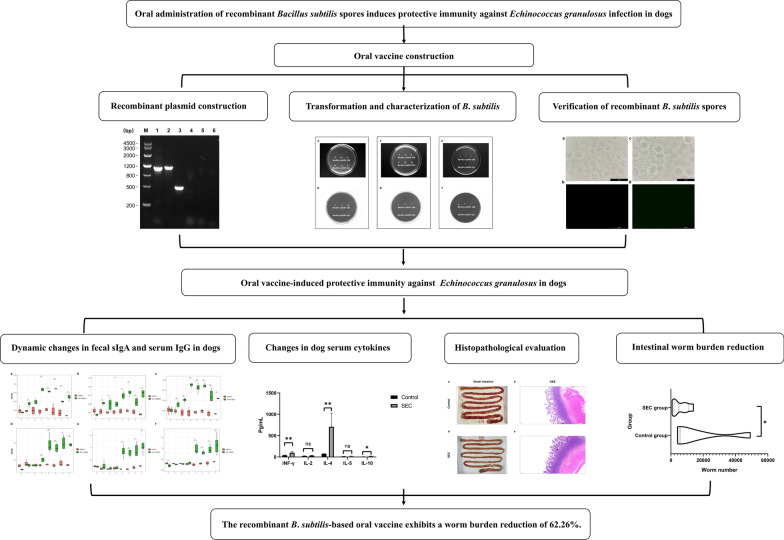

## Background

*Echinococcus granulosus* sensu lato is a canid intestinal cestode, whose metacestodes develop in the organs of livestock (e.g., sheep, cattle) and humans, causing cystic echinococcosis (CE) [1]. CE is a significant zoonotic disease that causes substantial economic losses in the livestock industry and poses serious public health threats in endemic regions [2]. Dogs are the definitive hosts of *E*. *granulosus* and the main reservoir of CE transmission. Humans and animals are typically infected by ingesting food or water contaminated with fecal eggs from infected dogs [3]. Currently, praziquantel-based deworming remains the cornerstone strategy for interrupting transmission by targeting the definitive host [4, 5]. However, its large-scale implementation is often impractical because of the need for frequent administration (at least once every 3 months), which is rarely achieved in endemic regions [6, 7]. Therefore, alternative preventive strategies—such as a vaccine capable of effectively reducing intestinal parasite burden in dogs—are critical for controlling CE transmission.

Substantial progress has been made with parenteral vaccines against *E*. *granulosus*, especially EgM-family proteins (notably EgM123), which have shown > 90% protection [8]. Other promising candidates include recombinant 3-hydroxyacyl-CoA dehydrogenase [9] and the EgTIM/EgANXB3 cocktail vaccine [10]. Oral vaccination offers practical advantages over parenteral administration, especially for large-scale canine immunization in resource-limited regions. To date, only one oral vaccine candidate has been tested in challenge trials: a *Salmonella typhimurium* vaccine expressing the EgA31-EgTrp fusion antigen, which achieved a 70–80% worm burden reduction (adjusted to 51–62% compared with *S*. *typhimurium* vector control) [11]. The observed efficacy gap between oral and parenteral vaccines underscores the need for improved antigen selection and more effective oral delivery vectors.

*Bacillus subtilis*, a well-characterized gram-positive bacterium, has great potential for oral antigen delivery because of its robust endospore formation and probiotic compatibility in mammalian hosts [12, 13]. Heterologous protein expression on the *B*. *subtilis* spore surface enables stable antigen presentation [14]. This strategy involves genetic fusion of antigens to native spore-coat proteins (e.g., CotB or CotC), allowing durable antigen display during sporulation while preserving structural integrity [15]. Previous studies have shown that recombinant *B*. *subtilis* spores displaying *E*. *granulosus* tropomyosin/paramyosin fusion antigens can elicit long-lasting immune responses [14, 16]. These findings support the use of *B*. *subtilis* spores as a promising live vector for *E*. *granulosus* antigen delivery.

Building on prior studies, this work identified *E*. *granulosus* enolase (EgENO), severin (EgSev), and cyclophilin (EgCyc) as new candidate antigens to develop an oral vaccine against canine *E*. *granulosus* infection. *Bacillus* *subtilis* spores were used as an oral delivery system to express EgSev, EgENO, and EgCyc fused to the CotB spore-coat protein. A cocktail of the three recombinant spores was orally administered to dogs, followed by challenge experiments to evaluate vaccine efficacy.

## Methods

### Animals, parasites, and biological materials

Twelve 2-year-old beagles (6 males and 6 females) were obtained from the Beagle Breeding Center, Sichuan Institute of Musk Deer Breeding (Sichuan, China). All animals were maintained under specific pathogen-free conditions in a controlled quarantine facility and fed a standardized commercial diet with ad libitum access to tap water.

Hydatid cysts were obtained from the livers of naturally infected sheep slaughtered at an abattoir in Sichuan Province, China. Protoscoleces (PSCs) were isolated from the cysts according to protocols detailed in our previous study [17]. The PSC suspension and 0.4% trypan-blue solution (Solarbio, Beijing, China) were mixed at a 9:1 (v/v) ratio (final concentration 0.04%). After staining for 3 min, PSC viability was evaluated under the microscope. PSCs that appeared blue were classified as dead, whereas unstained PSCs were considered viable. Only PSCs with viability > 95% were used for vaccination trials. Species identification was confirmed by PCR amplification and sequencing, identifying *E*. *granulosus* sensu stricto.

*Bacillus* *subtilis* strain 168 was purchased from Beijing Zoman Biotechnology Co., Ltd. (Beijing, China). The recombinant plasmid pDG364-CotB and purified recombinant proteins expressed in the *Escherichia coli* system (rEgSev, rEgENO, rEgCyc) were kindly provided by the Parasitology Research Center, Sichuan Agricultural University, Sichuan, China.

### Plasmid construction

Primers with restriction enzyme recognition sites (*Hind*III/*EcoR*I) were designed using Primer Premier 5.0 (PREMIER Biosoft, USA) based on target sequences obtained from GenBank (Table 1). PCR amplification of the *EgSev*, *EgEno*, and *EgCyc* genes was performed using a mixed cDNA template derived from *E*. *granulosus* PSCs and adult worms. Amplicons were verified via electrophoresis using a 1% agarose gel. Bands of the expected sizes were purified using a TIANgel Purification Kit (Tiangen, China), following the manufacturer’s protocol. The vector pDG364-CotB and the purified PCR products (*EgSev*, *EgEno*, *EgCyc*) were double-digested with *Hind*III and *EcoR*I restriction enzymes. The digested products were separated by agarose gel electrophoresis, and bands corresponding to the linearized vector and gene inserts were purified using the same protocol. The digested *EgSev*, *EgEno*, and *EgCyc* DNA fragments were each ligated into the *Hind*III/*EcoR*I-digested pDG364-CotB vector using T4 DNA ligase (Takara Bio, Japan). The recombinant plasmids were named pDG364-CotB-EgSev, pDG364-CotB-EgENO, and pDG364-CotB-EgCyc. The recombinant plasmids were verified by PCR using three primer sets: CotB-F/R (spanning the full spore-coat gene), CotB-F/gene-specific-R (insert-vector junction), and gene-specific-F/R (insert gene region). The PCR products were sequenced by Sangon Biotech (Shanghai, China) to confirm the correct insertion. Verified recombinant plasmids were stored at –80 °C for further experiments.

**Table 1:** Names and sequences of PCR primers used for constructing recombinant vectors

Gene	Primers	Reference sequences	Primer sequences (5’ → 3’)	Restriction endonuclease	Expected size without italicized sequence (bp)
*EgEno*	F-ENO	GU080332.1	CC*AAGCTT* TCCATCTTAAAGATCCATGCCCG	*Hind*III	1302
	R-ENO		CG*GAATTC* TTACAAAGGATTGCGGAAGTGCTCG	*Eco*RI	
*EgSev*	F-Sev	XM_024497312.1	CC*AAGCTT* GCGGGTCTTGTGAAAGC	*Hind*III	1101
	R-Sev		CG*GAATTC* CTACTCATCCCAAATGCCCTTT	*Eco*RI	
*EgCyc*	F-Cyc	AF430707.1	CC*AAGCTT* GGCGTGAAGTGCTTC	*Hind*III	489
	R-Cyc		CG*GAATTC* TTACAGCTGACCGCAGTCAGTAATC	*Eco*RI	
*CotB*	F- CotB	UVZ46003.1	CG*GGATCC* ACGGATTAGGCCGTTTGTCC	*Bam*HI	1104
	R- CotB		CC*AAGCTT* GGATGATTGATCATCTGAAG	*Hind*III	

### Transformation of ***B. subtilis***

The recombinant plasmids (pDG364-CotB-EgSev, pDG364-CotB-EgENO, and pDG364-CotB-EgCyc) were linearized using *Xba*I restriction enzyme digestion. Each linearized plasmid was introduced into competent *B*. *subtilis* strain 168 cells following the method of Cutting and Vander Horn [18]. After transformation, bacterial suspensions were plated on Luria-Bertani (LB) agar containing 5 μg/ml chloramphenicol. Plates were incubated in an inverted position at 37 °C overnight. Single colonies were selected and cultured in 5 ml of LB broth supplemented with 5 μg/ml chloramphenicol. Cultures were incubated at 37 °C for 16 h. Recombinant strains were named *B*. *subtilis* EgSev, *B*. *subtilis* EgENO, and *B*. *subtilis* EgCyc.

### Amylase activity assay and spore preparation

One microliter of each recombinant *B*. *subtilis* culture was spotted onto LB agar plates supplemented with 1% soluble starch. The wild-type *B*. *subtilis* 168 strain was used as a negative control. Plates were incubated overnight at 37 °C. Amylase activity was visualized by flooding the plates with iodine solution, which reacts with starch. Recombinant *B*. *subtilis* spores were prepared and purified following the protocol described by Vogt et al. [14].

### Indirect immunofluorescence assay

To verify antigen surface expression on recombinant *B*. *subtilis* spores, an indirect immunofluorescence assay (IFA) was conducted. Purified spore suspensions were fixed onto clean glass slides. The slides were blocked with 3% bovine serum albumin (BSA) in phosphate-buffered saline (PBS) for 30 min at room temperature. The slides were then washed three times with PBS for 3 min per wash. The primary antibody (serum from a *E*. *granulosus*-infected dog, diluted 1:100 in 1% BSA) was added to the slides. The slides were incubated at room temperature for 1 h, followed by three PBS washes (3 min each). The slides were incubated in the dark with a secondary antibody solution (Rabbit Anti-Dog IgG/FITC), Solarbio, China; 1:500 in 1% BSA) for 45 min at room temperature. After incubation, the slides were washed three times with PBS (3 min per wash). Fluorescence signals were examined and imaged using a fluorescence microscope.

### Oral immunization and parasite challenge

**Fig. 1:**
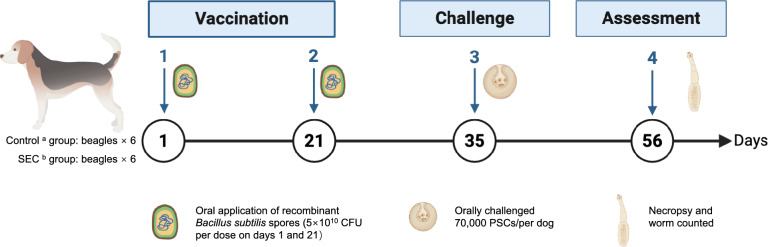
The workflow of the study, highlighting the vaccination protocol, timing of parasite challenge, and evaluation of vaccine efficacy in two experimental groups. ^a^Control group: each dog received three doses of 5 × 10^10^ colony-forming units (CFU) of recombinant *Bacillus subtilis* 168 spores. ^b^SEC group: each dog was orally administered a cocktail of recombinant spores, including *B. subtilis* EgSev, *B. subtilis* EgENO, and *B. subtilis* EgCyc

Twelve beagles were randomly divided into two groups (*n* = 6). The immunization and challenge schedules are illustrated in Fig. 1. Control group dogs received 3 × 5 × 10^10^ colony-forming units (CFU) of wild-type *B*. *subtilis* 168 spores mixed with food at each immunization time point. Dogs in the vaccinated group (SEC group) were orally immunized with a cocktail vaccine consisting of equal proportions of *B*. *subtilis* EgSev, EgENO, and EgCyc spores (5 × 10^10^ CFU each), administered with food. Each dog received two oral immunizations: a primary immunization on Day 0 (D0) and a booster on Day 21 (D21). On Day 35 (D35), all dogs were orally infected with 70,000 PSCs. On Day 56 (D56), the dogs were humanely killed for necropsy. The dog’s small intestine was carefully removed and opened longitudinally. The intestinal segment was then incubated in PBS at 37 °C for 1 h to allow parasites to detach. The supernatant was discarded, and the remaining fluid containing segmented worms was repeatedly replaced with fresh PBS until the suspension was clear. Worms present in the fluid were then counted under a microscope to determine the total parasite burden. Tissue samples from the small intestine were fixed in 4% paraformaldehyde for histological analysis. Serum and fecal samples were collected weekly to assess serum IgG, cytokine, and fecal secretory IgA (sIgA) levels.

### Indirect ELISA for sIgA and IgG

Fecal sIgA and serum-specific IgG levels were measured using an indirect enzyme-linked immunosorbent assay (ELISA). The ELISA plates (96-well) were coated with recombinant rEgENO, rEgSev, or rEgCyc proteins (5 µg/ml in 0.05 M carbonate-bicarbonate buffer, pH 9.6) at 100 µl/well and incubated overnight at 4 °C. The plates were washed three times with PBST (0.05% Tween-20 in PBS; 200 µl/well; 5 min each). The wells were blocked with 5% (w/v) skim milk in PBS and incubated at 37 °C for 2 h. After washing, serum (1:100 in PBST) or fecal samples (1:5 in PBST) were added and incubated at 37 °C for 1 h. Following another wash, HRP-conjugated rabbit anti-dog IgG (1:3000, Solarbio, China) or HRP-conjugated goat anti-canine IgA (1:10,000, Novusbio, USA) was added to detect serum IgG and fecal sIgA, respectively, and incubated for 1 h at 37 °C. The plates were washed and developed using 3,3′5,5′- tetramethylbenzidine (TMB) substrate solution. The plates were incubated at room temperature in the dark for 15 min. The reaction was stopped using 2 M H₂SO₄. The optical density (OD) was then measured at 450 nm using a microplate reader.

### Serum cytokine levels

Serum concentrations of interleukin-2 (IL-2), interleukin-4 (IL-4), interleukin-5 (IL-5), interleukin-10 (IL-10), and interferon-gamma (IFN-γ) were measured using commercial Canine IL-2, IL-4, IL-5, IL-10, and IFN-γ ELISA kits, respectively (Solarbio, China). All reagents were equilibrated to room temperature before use. The assays were conducted in accordance with the manufacturer’s protocols.

### Histopathological analysis

Small intestinal tissue samples from dogs were processed following standardized histological procedures. Intestinal segments (1 cm^3^) were immediately fixed in 4% paraformaldehyde (0.1 M PBS, pH 7.4) at 4 °C for 24 h, with one buffer change. Fixed samples were dehydrated in a graded ethanol series, cleared with xylene, and embedded in paraffin at 56–58 °C. Paraffin-embedded tissues were sectioned at 5 μm, stained with H&E (hematoxylin for 5 min, eosin for 2 min) and PAS (0.5% periodic acid for 10 min, Schiff's reagent for 15 min), and then mounted with neutral resin. Three serial sections were prepared per tissue sample.

### Statistical analysis

Vaccine protection efficacy was calculated using the formula:

Mean worm burden in the control group—Mean worm burden in the vaccinated group)/Mean worm burden in the control group × 100%. Statistical analysis was performed using the F-test, and differences were considered significant at *P* < 0.05. Data were analyzed using SPSS 20.0 (IBM Corp., USA) and GraphPad Prism 8.0 (GraphPad Inc., USA).

## Results

### PCR amplification

**Fig. 2:**
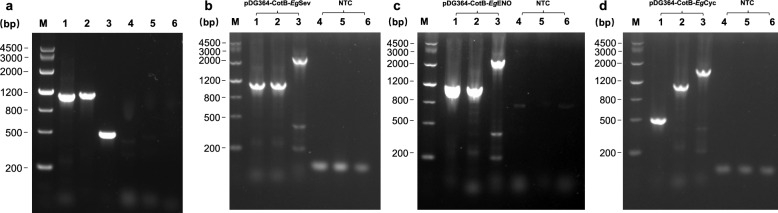
PCR amplification of target genes and verification of recombinant plasmids. **a** PCR amplification of *Echinococcus* *granulosus* target genes from mixed cDNA of PSC and adult worms. Specific bands were detected for *EgSev* (1101 bp, lane 1), *EgEno* (1302 bp, lane 2), and *EgCyc* (489 bp, lane 3). Lanes 4–6 represent no-template controls (NTCs) for the respective primer sets. M: DNA marker. **b–d** PCR-based verification of recombinant plasmids pDG364-CotB-EgSev, pDG364-CotB-EgENO, and pDG364-CotB-EgCyc. For pDG364-CotB-EgSev (**b**), amplification using primer pairs F-EgSev/R-EgSev (1101 bp, lane 1), F-CotB/R-CotB (1104 bp, lane 2), and F-CotB/R-EgSev (2205 bp, lane 3) confirmed correct construct assembly. For pDG364-CotB-EgENO (**c**), amplification with F-EgENO/R-EgENO (1302 bp, lane 1), F-CotB/R-CotB (1104 bp, lane 2), and F-CotB/R-EgENO (2406 bp, lane 3) yielded expected bands. For pDG364-CotB-EgCyc (**d**), amplification using F-EgCyc/R-EgCyc (489 bp, lane 1), F-CotB/R-CotB (1104 bp, lane 2), and F-CotB/R-EgCyc (1593 bp, lane 3) produced the anticipated products. Lanes 4–6 in each panel represent the corresponding NTCs

The *EgEno* (1302 bp), *EgSev* (1101 bp), and *EgCyc* (489 bp) cDNAs were successfully amplified from mixed PSC/adult worm cDNA (Fig. 2a). Sanger sequencing confirmed that all PCR products were 100% identical to their corresponding sequences in the GenBank database (Table 1).

### Verification of recombinant plasmids

The *EgSev*, *EgEno*, and *EgCyc* cDNAs were successfully inserted into the pDG364-CotB vector, generating the recombinant plasmids pDG364-CotB-EgSev, pDG364-CotB-EgENO, and pDG364-CotB-EgCyc. PCR analysis yielded products of the expected sizes for each construct plasmid (Fig. 2b-d). Sanger sequencing confirmed the correct insert orientation and sequence identity.

### Construction of recombinant *B*. *subtilis* and characterization of amylase activity

**Fig. 3:**
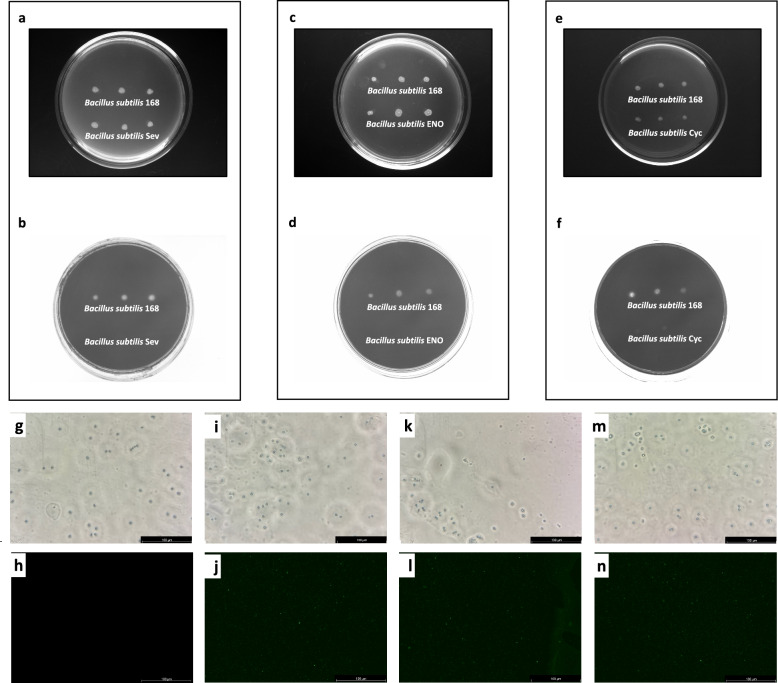
Identification of recombinant *Bacillus subtilis* strains and spores. **a–f** Identification of recombinant *B*. *subtilis* strains by amylase activity assay. **a**, **c**, **e** Growth of *B*. *subtilis* 168 and recombinant strains (*B*. *subtilis* EgSev, *B*. *subtilis* EgENO, and *B*. *subtilis* EgCyc, respectively) on LB agar plates containing 1% starch. **b**, **d**, **f** Starch hydrolysis visualized by iodine staining of the corresponding plates, indicating amylase activity. **g**–***n*** Identification of recombinant *B*. *subtilis* spores by immunofluorescence analysis. **g**, **h** Bright-field (**g**) and immunofluorescent (**h**) images of *B*. *subtilis* 168 spores. **i**, **j** Bright-field (**i**) and immunofluorescent (**j**) images of *B*. *subtilis* EgSev spores. **k**, **l** Bright-field (**k**) and immunofluorescent (**l**) images of *B*. *subtilis* EgENO spores. **m**, ***n*** Bright-field (**m**) and immunofluorescent (***n***) images of *B*. *subtilis* EgCyc spores

The recombinant plasmids were introduced into *B*. *subtilis* 168 genomes via homologous recombination, integrating the fusion genes at the *AmyE* locus. Wild-type *B*. *subtilis* 168 colonies produced a distinct halo of starch hydrolysis caused by amylase activity (Fig. 3a–f, upper line). Loss of amylase activity, indicated by absence of starch hydrolysis halos (Fig. 3a–f, lower line) around the recombinant *B*. *subtilis*, confirmed successful gene insertion.

### IFA of recombinant B. subtilis spores

Spores of the recombinant *B*. *subtilis* strains were successfully prepared and purified. According to the IFA results, antigen-specific fluorescence signals on the surface of *B*. *subtilis* EgSev (Fig. 3i, j), EgENO (Fig. 3k, l), and EgCyc (Fig. 3m, n) spores were observed, but not in the wild-type controls (Fig. 3g, h), indicating successful surface display of the antigens.

### Induction of protective immunity against *E. granulosus* infection in dogs

Three weeks after oral challenge with PSCs, dogs from both the SEC and control groups were humanely killed for necropsy. The worm burdens for each group are summarized in Table 2. Statistical analysis revealed a significant reduction in the intestinal worm burden in the SEC-vaccinated group compared with that of the controls. The mean worm burden [± standard error of the mean (SEM)] was 16,233 ± 8633 in the control group and 6126 ± 2097 in the SEC group. This represents a worm burden reduction of 62.26% in the SEC group (*P* < 0.05; Table 2).

**Table 2:** Intestinal worm burden and reduction rates in dogs following oral immunization with recombinant *Bacillus subtilis* spores expressing *Echinococcus granulosus* antigens

Group	*B*. *subtilis* spores applied	Dog no	Number of worms	Average	Reduction (%)	*P* value
Control group	3 × 5 × 10^10^ CFU	Dog-BS-1	49,166	16,233	–	–
		Dog-BS-2	5666			
		Dog-BS-3	4166			
		Dog-BS-4	18,000			
		Dog-BS-5	4166			
		Dog-BS-6	-			
SEC group	5 × 10^10^ CFU EgSev 5 × 10^10^ CFU EgENO 5 × 10^10^ CFU EgCyc	Dog-BS-7	2333	6126	62.26%	< 0.01
		Dog-BS-8	760			
		Dog-BS-9	2666			
		Dog-BS-10	9833			
		Dog-BS-11	7166			
		Dog-BS-12	14,000			

### Dynamic changes in fecal sIgA, serum IgG, and cytokines

**Fig. 4:**
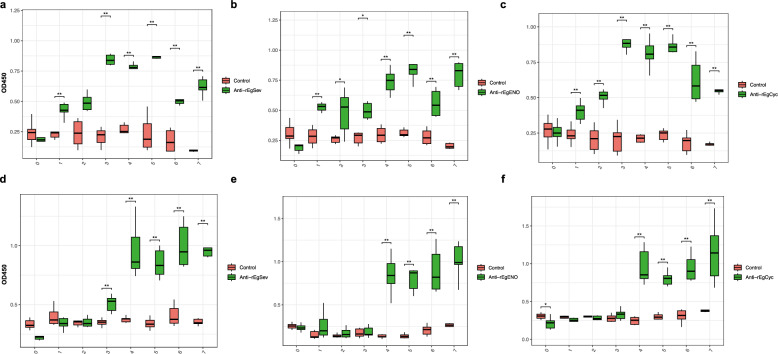
Fecal sIgA and serum IgG levels following oral immunization. **a–c** OD 450 values of fecal secretory IgA (sIgA) after oral immunization with *Bacillus subtilis* EgSev (**a**), *B. subtilis* EgENO (**b**), and *B. subtilis* EgCyc (**c**). **d**–**f** OD 450 values of serum IgG after oral immunization with *B. subtilis* EgSev (**d**), *B. subtilis* EgENO (**e**), and *B. subtilis* EgCyc (**f**). Red bars represent samples from the control group, while green bars represent samples from the SEC group. *P < 0.05, **P < 0.01, ***P < 0.001

Dogs immunized with SEC showed an increase in antigen-specific fecal sIgA levels (Fig. 4a–c) for rEgSev, rEgENO, and rEgCyc as early as 1 week after the primary immunization (D7), indicating induction of mucosal immunity. After the booster immunization (D21), sIgA levels increased significantly (P/N ≥ 2.1, *P* < 0.05 vs. control), although marked individual variability was observed.

Antigen-specific serum IgG levels in the SEC-immunized dogs increased significantly 1 week after booster immunization (D28) (Fig. 4d–f), showing a delayed response relative to sIgA. Peak IgG titers against all three antigens were observed at D28 in the SEC-vaccinated dogs and remained significantly elevated through D56 (*P* < 0.05 compared with the control).

**Fig. 5:**
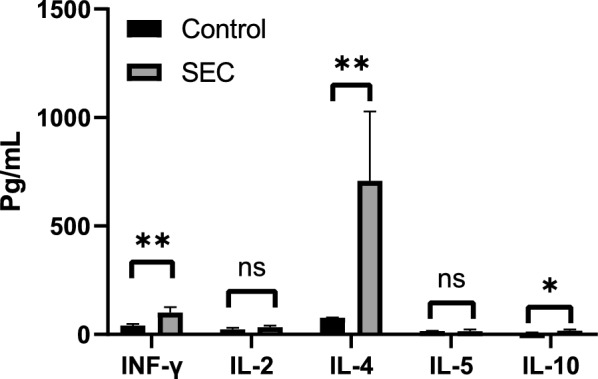
Cytokine response profiles 2 weeks after booster immunization. In the SEC group, levels of interleukin (IL)-4, IL-10, and interferon gamma (IFN-γ) were significantly elevated at day 14 post-immunization, while IL-2 and IL-5 levels remained unchanged. *P < 0.05, **P < 0.01, ***P < 0.001

By day 14 after booster immunization (D35), serum cytokine profiling (Fig. 5) revealed that levels of IFN-γ, IL-4, and IL-10 in the vaccinated group were significantly higher than those in the control group (*P* < 0.05). The marked increase in IFN-γ at D35 (*P* < 0.05) suggested the involvement of the IFN-γ-mediated pathway in reducing the parasite load.

### Histopathological analysis

**Fig. 6:**
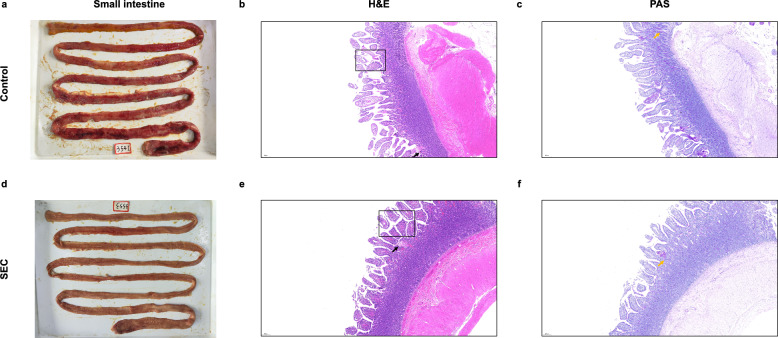
Histopathological analysis of the small intestine on day 21 post-*Echinococcus granulosus* challenge. **a** Representative small intestine tissue collected from a dog in the control group. **b–c** Tissue sections stained with hematoxylin and eosin (H&E) (**b**) and periodic acid-Schiff (PAS) (**c**). **d** Representative small intestine tissue collected from a dog in the SEC-vaccinated group. **e–f** Corresponding sections stained with H&E (**e**) and PAS (**f**). Parasite attachment sites, hemorrhagic exudates, and goblet cells are indicated by black frames, black arrows, and yellow arrows, respectively. Scale bar: 200 μm

Histopathological evaluation demonstrated that SEC vaccination conferred protection against *E*. *granulosus*-induced intestinal injury (Fig. 6a–f). Dogs in the control group exhibited extensive intestinal edema, accompanied by bloody exudates adhering to the mucosal surface (Fig. 6b). By contrast, dogs in the SEC-vaccinated group displayed well-preserved mucosal architecture (Fig. 6e). All groups showed strong PAS-positive staining in the intestinal mucosa (Fig. 6c, f). Notably, the goblet cell density was higher in the control tissues than in the SEC group, suggesting that parasite colonization might have triggered compensatory mucus hypersecretion.

## Discussion

Cystic echinococcosis, caused by the larval stage of *E*. *granulosus* s.l., remains a major global public health concern, posing serious threats to human health and inflicting substantial economic losses on livestock industries [2, 19]. Dogs are the primary definitive host and main reservoir for human infection. The current strategy of monthly praziquantel deworming is hindered by high operational costs and the potential development of drug resistance, threatening its sustainability [5, 20]. These limitations highlight the urgent need to develop effective canine vaccines as complementary control tools.

Initially, canine vaccine studies focused on crude or ES antigens from PSCs and adult worms [20–22], showing protective responses. In recent years, several recombinant proteins have been investigated as vaccine candidates and were shown to reduce adult worm burden in dogs [20]. However, most recombinant protein vaccines are administered via injection, requiring physical restraint and trained personnel for proper delivery. Owing to the practical advantages of oral vaccination in dogs, several oral live-vector vaccines have been developed and successfully applied against diseases such as canine parvovirus [23], rabies [24], and respiratory infections [25]. To date, only two bacterial vectors, *S*. *typhimurium* and *B*. *subtilis*, have been evaluated to develop oral vaccines against *E*. *granulosus* in dogs. An oral vaccine based on *S*. *typhimurium* expressing the EgA31-EgTrp fusion protein achieved an adjusted > 51% worm burden reduction in dogs [11]. Additionally, although recombinant *B*. *subtilis* spores have been proposed as oral vaccine vectors, their protective efficacy in challenge models of canine *E*. *granulosus* infection remains unvalidated [14, 16]. Preliminary data suggest that oral vaccines are a promising approach for *E*. *granulosus* control in dogs; however, several critical challenges must be addressed. These include the optimization of antigen selection, confirmation of vector biosafety, and enhancement of antigen delivery efficiency.

To date, research on oral vaccines targeting *E*. *granulosus* in dogs has largely focused on the antigens EgA31 and EgTrp [11, 14, 16]. Other potential oral vaccine antigens have not been well studied. We identified three proteins exhibiting high potential as vaccine candidates [26]. EgENO is a critical glycolytic enzyme essential for parasite energy production. It is also a prominent component of adult worm derived exosomes and has been implicated in modulating host immune responses [27]. EgSev is located on the parasite tegument and is presumed to contribute to parasite motility, similar to the function proposed for EgA31 [28, 29]. EgCyc exhibits strong immunogenicity and its candidacy as a vaccine antigen is reinforced by consistent predictions across several bioinformatic platforms [30]. Based on these findings, EgSev, EgENO, and EgCyc were selected as target antigens for the formulation of a cocktail oral vaccine for dogs in this study.

Building on the established oral vaccine delivery systems against *E*. *granulosus* in dogs, this study selected *B*. *subtilis* as the oral vaccine vector. First, regarding biosafety, the *B*. *subtilis* spore-based oral delivery platform provides notable advantages in both safety and ease of administration [31]. Throughout the entire experimental period, orally immunized dogs exhibited no clinical signs of adverse reactions. Histopathological analysis revealed no notable pathological changes in the intestinal tissues of the vaccinated dogs. Moreover, the immunized dogs showed histological evidence of intestinal tissue repair from damage usually induced by *E*. *granulosus* infection. Furthermore, *B*. *subtilis* is classified as GRAS (Generally Recognized As Safe) and exhibits probiotic characteristics [32]. It is also a natural commensal microorganism in the canine intestinal tract, further supporting its safety profile as an oral delivery vector [33, 34]. Second, in terms of antigen delivery, *B*. *subtilis* spores possess intrinsic resistance to gastric acid, thus preserving the structural integrity of the displayed antigens and enabling effective delivery to the intestinal mucosa [16, 35]. Both prior and current findings confirm that recombinant spores displaying fusion antigens are capable of eliciting robust humoral immune responses. Importantly, upon germination in the gut, spores activate gut-associated lymphoid tissue, thereby facilitating prolonged immune system stimulation [16]. Third, regarding efficacy, the SEC vaccine group exhibited a 62.26% reduction in the worm burden following oral immunization, demonstrating considerable vaccine efficacy. This outcome represents an approximately 10% improvement over previous oral vaccine candidates, highlighting the delivery efficacy of *B. subtilis* spores [11, 20].

Notably, although the oral vaccine evaluated in this study elicited substantial efficacy, marked inter-individual variation was observed among the vaccinated dogs. Such heterogeneity in immune response has also been noted in other studies of oral vaccines [11, 36]. This variability was manifested in the antibody responses. Compared with injectable vaccines, oral immunization may be more prone to individual or administration-related variation, because the vaccine must traverse the gastrointestinal tract and mucosal immune system and is subject to degradation by gastric acid, digestive enzymes, and the intestinal environment, thereby increasing the likelihood of low responders [16, 37]. A high proportion of low responders might compromise the establishment of effective herd immunity [38, 39]. Therefore, further investigation is warranted to determine the underlying mechanisms of this immunological heterogeneity to improve the robustness and consistency of the vaccine-induced immune responses.

## Conclusions

The recombinant *B*. *subtilis*-based oral vaccine exhibited enhanced safety, efficient antigen delivery, and improved efficacy relative to currently available oral vaccines targeting *E*. *granulosus* in dogs. It effectively suppressed *E*. *granulosus* infection by significantly reducing the worm burden in the canine intestine. This vaccine could substantially improve the effectiveness of integrated CE control strategies in endemic regions. However, the variability in immune responses among vaccinated dogs, especially the occurrence of low responders, should be further investigated to guarantee herd immunity.

## Data Availability

All data generated or analyzed during this study are included in this published article.

## Abbreviations

*E*. *granulosus* s.l.: *Echinococcus granulosus* sensu lato
CE: Cystic echinococcosis
PSCs: Protoscoleces
IFA: Immunofluorescence assay
*S*. *typhimurium*: *Salmonella typhimurium*
*B*. *subtilis*: *Bacillus subtilis*
